# Editor’s introduction to this issue (G&I 20:1, 2022)

**DOI:** 10.5808/gi.20.1.e1

**Published:** 2022-03-31

**Authors:** Taesung Park

**Affiliations:** Department of Statistics, Seoul National University, Seoul 08826, Korea

In this issue, there are two review articles, eight original articles, one genome archive, and two application notes. In this editorial, I would like to focus on the two review articles, as well as two original articles and one application note on genome-wide association studies (GWAS).

Recent rapid advances in single-cell RNA sequencing have made it possible to recognize a variety of previously unidentified subpopulations and rare cell states in tumors and the immune system based on single-cell gene expression profiles. Single-cell RNA sequencing is the topic of the first review article, by Dr. Jong-Il Kim’s group (Seoul National University College of Medicine, Korea). This review addresses the current development of methods for constructing single-cell epigenomic libraries, including multi-omics tools with important elements and additional requirements for the future, focusing on DNA methylation, chromatin accessibility, and histone post-translational modification. Single-cell epigenomic libraries help to understand the principles of comprehensive gene regulation that determine cell fate through transcripts alone and the resulting output of gene expression programs. The corresponding single-cell epigenome is expected to elucidate the mechanisms involved in the origin and maintenance of a comprehensive single-cell transcriptome. This review insightfully summarizes current research trends in the field of cellular differentiation and disease development at the single-cell level, moving toward the single-cell epigenome.

The second review, led by Dr. Tung (Dagon University, Myanmar), deals with recent developments in whole-genome sequencing technologies. While the analysis of whole-genome sequencing data requires highly sophisticated bioinformatics tools, many researchers do not have the bioinformatics capabilities to analyze the genomic data and are therefore unable to take maximum advantage of whole-genome sequencing. This review provides a practical guide on a set of bioinformatics tools available online to analyze whole-genome sequence data of bacterial genomes and presents a description of their web interfaces.

Now, I would like to turn to three articles about GWAS. The main goal of GWAS is the identification of causal variants associated with the phenotype of interest. All GWAS introduce appropriate statistical models to explain the phenotype and then to perform statistical tests. An important challenge in this post-GWAS era is to increase statistical power by using better statistical models and tests, and to investigate the causal effects between modifiable risk factors and the phenotypes via Mendelian randomization (MR).

The first article, the first author of which is Dr. Wonil Chung (Soongsil University, Korea), is about Bayesian mixed models for longitudinal genetic data. The authors proposed a Bayesian variable selection method for longitudinal genetic data using mixed models. Joint modeling of the main effects and interactions of all candidate genetic variants along with non-genetic factors leads to improved statistical power. The authors provided the theoretical basis of the Bayesian method and evaluated its performance using data from the 1000 Genomes Project. By exploring various simulation settings, the authors showed that the proposed method tended to have higher statistical power than other existing methods. In particular, the proposed method was shown to detect well gene-time/environment interactions, which may account for some of the missing heritability.

The second article, written by Dr. Buhm Han (Seoul National University, Korea) and colleagues, is about improving the estimation of variance of causal effects in MR. When measuring causal effects between exposure and phenotype in GWAS, two-sample MR has been commonly used. The current two-sample MR uses a first-order approximation of standard error. Through simulation studies, the authors showed that this first-order approximation could lead to underestimation of variance of causal effects in MR. As a result, overestimation of power and an increased false-positive rate could occur. As an alternative, the authors proposed using the second-order approximation of the standard error to correct for the deviation of the first-order approximation and demonstrated its robust and accurate performance.

The third article on GWAS is about a supercomputing-aided approach (MPI-GWAS) to accelerate the permutation testing developed by Dr. Oh-Kyoung Kwon’s group (Korea Institute of Science and Technology Information, Korea). While permutation testing has the advantage of reducing inflated type 1 error rates, it suffers from high computational costs when applied to GWAS. With MPI-GWAS, it became possible to actionably compute a permutation-based GWAS in a reasonable amount of time, leveraging the power of parallel computing resources. I think that all three of these articles on GWAS will be good additions to the state-of-the-art knowledge on GWAS methodology.

